# Inflammatory papillary hyperplasia: A systematic review

**DOI:** 10.4317/medoral.21405

**Published:** 2016-12-06

**Authors:** Patricia Gual-Vaqués, Enric Jané-Salas, Sonia Egido-Moreno, Raúl Ayuso-Montero, Antoni Marí-Roig, José López-López

**Affiliations:** 1 DDS, Master’s degree. School of Dentistry, University of Barcelona. University Campus of Bellvitge, Barcelona, Spain; 2 DDS, MD, PhD. Department of Odontoestomatology. School of Dentistry, University of Barcelona. University Campus of Bellvitge, Barcelona, Spain. / Oral Health and Masticatory System Group (Bellvitge Biomedical Research Institute) IDIBELL, Barcelona, Spain; 3 DDS, Professor of Master’s degree. School of Dentistry, University of Barcelona. University Campus of Bellvitge, Barcelona, Spain; 4 DDS, MD, PhD, Specialist in Maxillofacial Surgery. Head of Department of Maxillofacial Surgery, University Hospital of Bellvitge. Catalonia, Spain. / Oral Health and Masticatory System Group (Bellvitge Biomedical Research Institute) IDIBELL, Barcelona, Spain

## Abstract

**Introduction:**

Inflammatory papillary hyperplasia (IPH) is a benign lesion of the palatal mucosa. It is usually found in denture-wearers but also has been reported in patients without a history of use of a maxillary prosthesis use.

**Objetives:**

The aim of this study is to review the literature to assess the prevalence of denture stomatitis and inflammatory papillary hyperplasia and the etiological factors associated.

**Material and Methods:**

A search was carried out in PubMed (January 2005 to October 2015) with the key words *“inflammatory papillary hyperplasia”, “denture stomatitis”, “granular stomatitis” and “Newton’s type III”* The inclusion criteria were studies including at least a sample of 50 apparently healthy patients, articles published from 2005 to 2015 written in English. The exclusion criteria were reviews and non-human studies.

**Results:**

Out of the 190 studies obtained initially from the search 16 articles were selected to be included in our systematic review. The prevalence of denture stomatitis was 29.56% and 4.44% for IPH. We found 5 cases of denture stomatitis among non-denture-wearer individuals. All IPH cases were associated with the use of prosthesis. Smoking and continued use of ill-fitting dentures turned out to be the most frequent risk factors for developing IPH.

**Conclusions:**

IPH is a rare oral lesion and its pathogenesis still remains unclear. Its presentation among non-denture-wearers is extremely unusual.

**Key words:**Inflammatory papillary hyperplasia, denture stomatitis, prevalence, granular stomatitis, Newton’s type III stomatitis.

## Introduction

**Figure 1:**
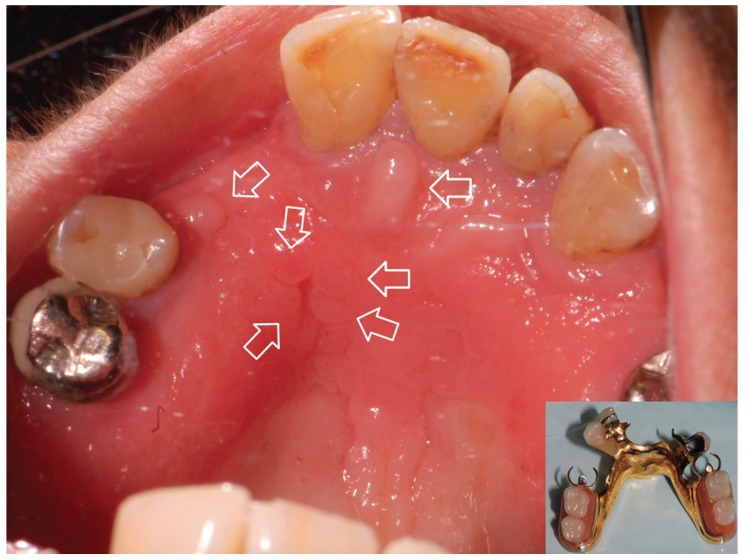
Inflammatory papillary hyperplasia. A case showing some injuries (marked by arrow) located under the framework.

Inflammatory papillary hyperplasia (IPH) is a benign lesion of the oral mucosa which is characterized by the growth of one or more nodular lesions, measuring about 2mm or less (Fig. 1). The lesion almost exclusively involves the hard palate (1-4). Nevertheless, in rare instances, it also has been seen on the mandible (5). The lesion is mostly asymptomatic and the color of the mucosa may vary from pink to red (1-5).

In general, IPH is associated with the use of removable upper dentures although it also has been found in dentulous patients with no history of a dental prosthesis (1,5). The lesion can be classified in the third type of denture stomatitis (Newton’s classification, 1962) when it is related to removable prosthesis (6).

In a study carried out by Ettinger (7) with a sample of 700 subjects who were upper denture wearers, prevalence of IPH was found to be around 14%.

**Figure 2:**
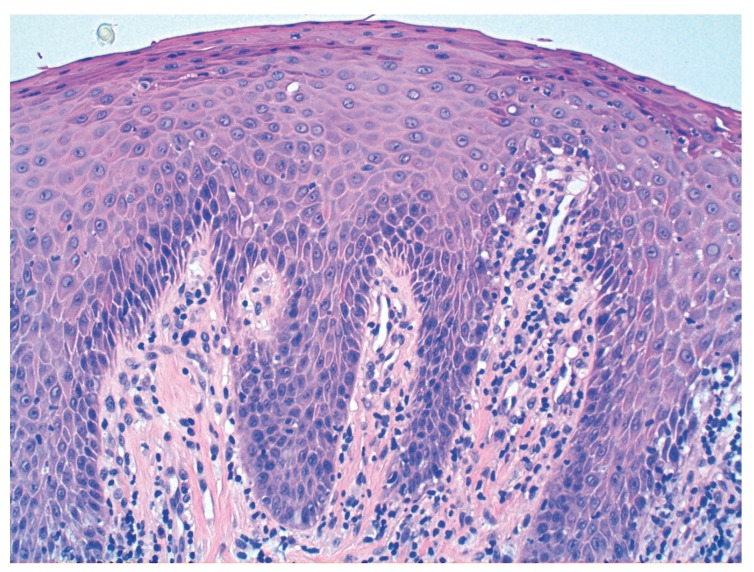
Epithelial hyperplasia with inflammation and fibrosis in Hematoxylin-Eosin at 200x magnification.

Histopathologically, the lesion has been described as papillary projections covered by stratified squamous epithelium with or without chronic inflammation (2) (Fig. 2). Although historically some authors believed the lesions had a premalignant component (1,5), the current predominant belief based on diverse and extensive histologic samples is that the lesion has a predominantly inflammatory nature (2,4).

The pathogenesis is unclear. The following have been suggested as potential etiological factors: ill-fitting dentures, continuous day and night denture use, poor oral hygiene, sensitivity to denture liners, tobacco, age related changes and some systemic conditions (1-4). IPH is also associated with the colonization of Candida caused by poor oral hygiene (4,5). However, Candida is not indispensable for the development of denture stomatitis (8-10).

The treatment of IPH varies widely among clinicians. The type of treatment rendered is related to the severity of the condition and the clinical presentation (4). When the clinical presentation is aggressive and large papillary lesions are present, clinicians have recommended laser (11), electrosurgery (12) or cryotherapy (13). Small localized lesions have been typically treated with mouthrinses. Orenstein & Taylor (4) use clorhexidine mouthrinse at 0.12% and Salonen *et al.* (14) prescribe antifungal mouthrinse or gels.

The objective of the present study was to evaluate the prevalence of denture stomatitis and inflammatory papillary hyperplasia in the past 10 years and the etiological factors associated with them.

## Material and Methods

In this systematic review, a search was carried out in MEDLINE (PubMed) database (January 2005 to October 2015) with the key words *“inflammatory papillary hyperplasia”, “denture stomatitis”, “granular stomatitis” and “Newton’s type III”*. The terms were merged using the Boolean operator “OR”. The inclusion criteria were studies including at least a sample of 50 apparently healthy patients, articles published from 2005 to 2015 written in English. The exclusion criteria were reviews and nonhuman studies. The articles were selected firstly by reading the titles and abstracts of the citations found in the bibliography to identify the most relevant studies and then, by means of reading the full-text article.

## Results

**Figure 3:**
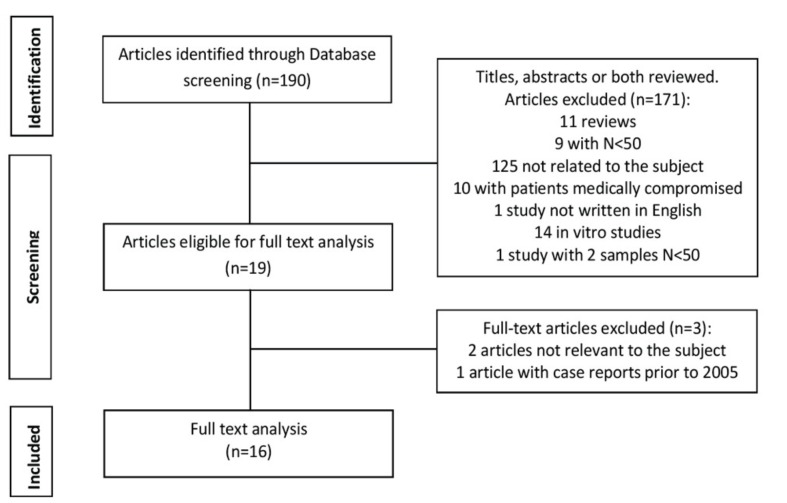
Flow of articles through the systematic review.

Out of the 190 studies obtained initially from the search 171 articles were excluded after applying the inclusion and exclusion criteria. Thus, the complete text of 19 studies was analyzed. Two of these 19 articles were excluded due to the lack of direct relationship with the subject and another one was removed because the sample included clinical cases from the year 1993 (Fig. 3).

The 16 articles were stratified according to their level of evidence, using Jadad scale (15). All of them were classified with a level 2 scientific evidence ( Table 1).

**Table 1:**
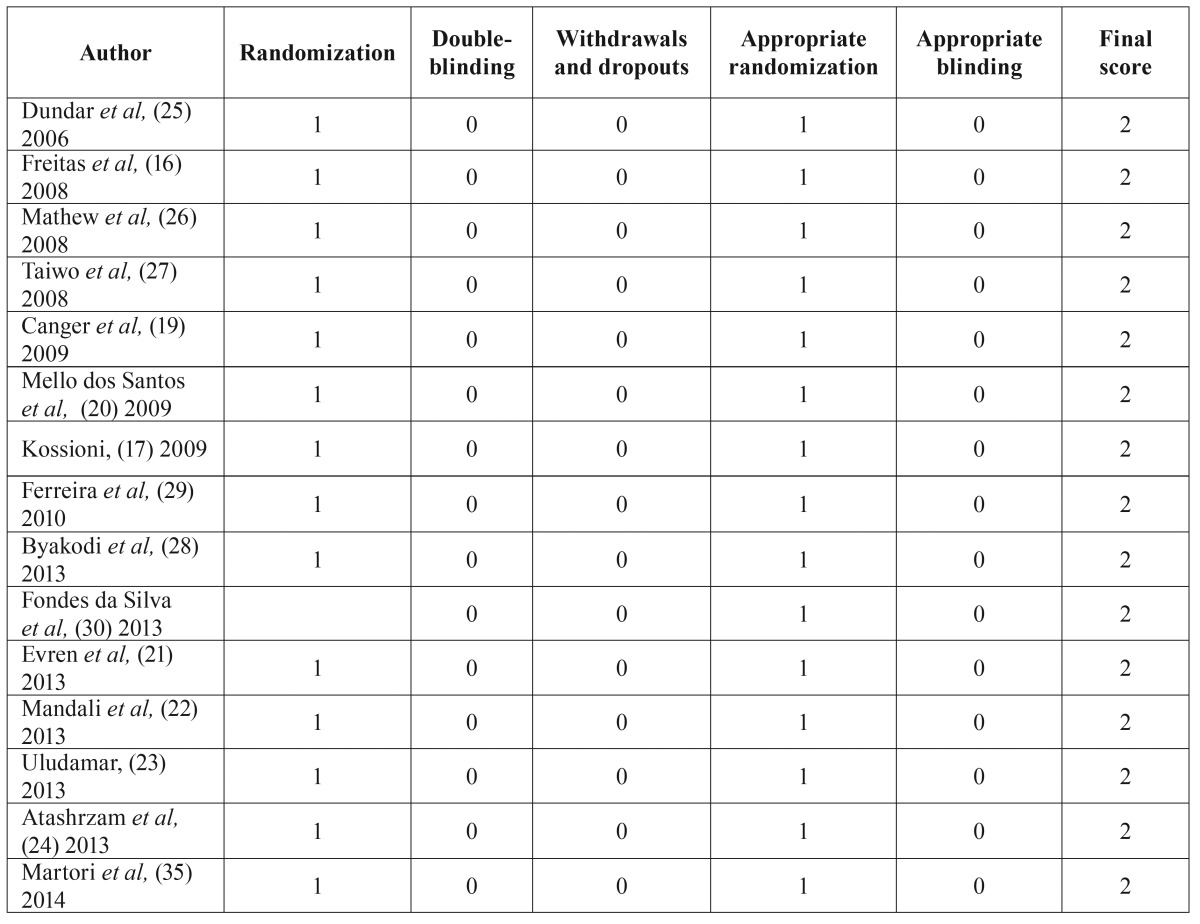
Quality assessment of the articles included in the review using Jadad scale (15).

The studies included analyzed, among other parameters, the prevalence of denture stomatitis in different population groups. Thus, with a total sample of 30.797 patients with a mean age ≥60 years, the prevalence of denture stomatitis for our review was found to be 29.56% ( Table 2).

**Table 2:**
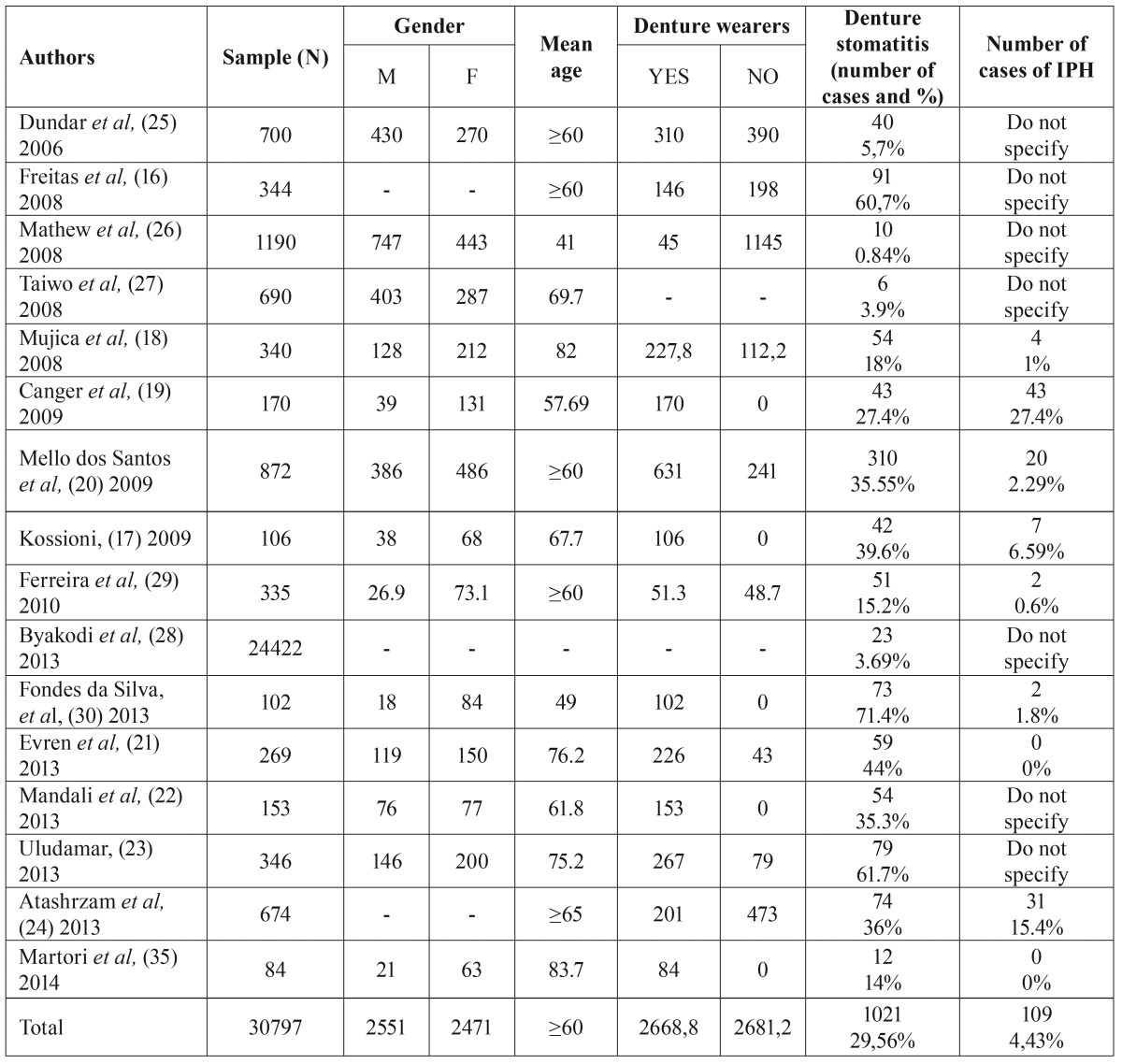
Summary of the 16 articles reviewed.

In the sample analyzed, all cases of papillary inflammatory hyperplasia (109 cases) occur in denture wearers, with a prevalence of 4.43%. Freitas *et al.* (16) reported 5 cases of denture stomatitis in non-denture-wearing patients. Nevertheless, the authors did not specify the type of denture stomatitis according to Newton’s classification (6). So, we can conclude that the prevalence of IPH in non- denture-wearing patients for our sample is 0% ( Table 3).

**Table 3:**
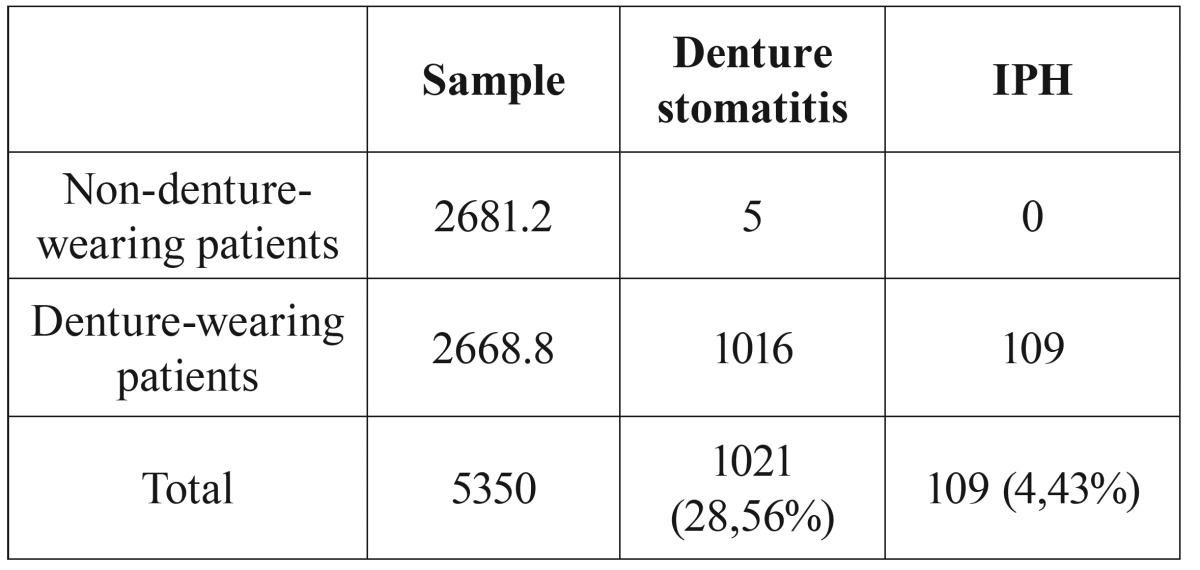
Prevalence of IPH.

## Discussion

The prevalence of denture stomatitis and the associated risk factors differ in various studies, mainly because of differences in research methodology (age of the study group, dental status of the study group, dental school patients or community population, institutionalized or non-institutionalized patients, different method of assessment of various factors and subjectivity of classification) (17).

In general, the prevalence rate of denture stomatitis is reported within the range of 20-67% (18), in accordance with the present review with a prevalence of 29.56%. The majority of the studies analyzed (16-24) report different rates of prevalence, all within this range. On the other hand (25-28), reported rates of prevalence of denture stomatitis lower than 6%. Mathew *et al.* (26), with a low prevalence of 0.84%, attributed the result to the low number of denture wearing subjects in the study group (45 out of 1190). According to Taiwo *et al.* (27), this observation is consistent with the finding of minimal tooth loss and edentulism in the study group.

Ferreira *et al.* (29) and Da Silva *et al.* (30) found a strong correlation between denture stomatitis and poor hygiene in the use of prostheses. This is in compliance with previous publications (31-34). Other factors, such as the continuous wearing of the dentures (16,17,19,20,25,26,28), and the integrity and adaptation of the prosthesis (16,18,19,28,35); have also been considered statistically significant factors.

Dundar *et al.* (25) found a correlation between diabetes mellitus and denture stomatitis. On the other hand, Kossioni (17) observed that denture stomatitis was more prevalent among smokers, even though the correlation was statistically insignificant.

The study of the prevalence of inflammatory papillary hyperplasia or type III denture stomatitis is complex because to the majority of publications fail to distinguish between different types of denture stomatitis, in accordance with Newton’s classification (6). In fact, in the literature reviewed we have not been able to find any article studying exclusively the IPH prevalence. The reality is that IPH was of great interest in the 60’s and 70’s when it was believed that the lesion had a premalignant component (1,5). This led to research and several studies were carried out in order to determine the cause and prevalence of the lesion [Ettinger, 1975 (7); Lambson, 1967 (36); Waite, 1961 (37)].

Out of the 16 articles reviewed only 9 mention inflammatory papillary hyperplasia. In the year 1975, Ettinger (7) assessed the prevalence of IPH in a sample of 700 subjects, obtaining a considerable prevalence of 13.9%. This result is in contrast with the occurrence rate found in our review (4.43%).

With regards to etiological factors, most of the literature suggests that there are several factors associated with IPH. Atashrazm *et al.* (24) reported 31 cases of IPH in a group of 201 complete denture wearers. The authors mention the presence of vacuum suction in all maxillary dentures and suggest a possible relationship with IPH.

With a prevalence of 2.29%, Dos Santos *et al.* (20) observed that patients with IPH were significantly most likely to be 70 years old or older, smokers and live in rural areas. In addition, the mean denture plaque score of those with IPH was higher. Al-Dwairi (38) also established a close relationship between smoking and IPH, suggesting that aggressive forms of granular stomatitis are associated with a daily consumption of 25 cigarettes. In compliance with previous studies (2,5), Canger *et al.* (19) concluded that the most significant risk factor for IPH is wearing ill-fitting dentures for more than 10 years. In addition, and in accordance to Coelho *et al.*(39), gender was also found to be another significant factor. The high prevalence of IPH within the females can be attributed to the fact that females live longer than men, more women wear dentures (and for longer periods) than men and also due to postmenopausal changes that make the oral mucosa more susceptible to hyperplasic changes.

In compliance with Fisher *et al.* (2), Kossioni (17) noted that the frequency of denture cleaning was significantly related to IPH. Other factors such as age, gender and tobacco were not found to be significant for any type of denture stomatitis.

In the literature reviewed, we have found only 2 cases of IPH in non-denture-wearing patients. The first case, published in 1976, refers to a dentulous patient who had never worn dental prosthesis. On the oral examination, multiple papules could be seen on the palatal mucosa. The authors concluded that there was no apparent reason for occurrence of IPH in this individual (5). The second case report presented a 10 year old female who had a single nodule on the hard palate. The oral examination revealed poor oral hygiene conditions and mouth breathing. The child had no history of appliance therapy with either a metal or acrylic base. An excisional biopsy of the lesion was done and the provisional diagnosis of IPH was thus confirmed. In this case, the authors attributed the IPH to the poor oral hygiene and the fact the patient was a mouth breather (4).

Conclusions

In compliance with the literature reviewed, the prevalence of denture stomatitis varies from 20-67%. Poor oral hygiene and the continuous use of dentures were found to be the most significant risk factors for developing denture stomatitis.

IPH is a rare benign lesion and its pathogenesis remains unclear. The predisposing factors most widely described are continuous day and night ill-fitting denture use, poor oral condition and smoking.

The presence of IPH among non-denture-wearing patients is extremely unusual although is more likely to occur in patients with poor oral hygiene, smokers and/or mouth breathers.

In short, we would like to emphasize that a maxillary denture may not be the exclusive etiology for the appearance of IPH.
